# Social cure model: testing the link between identity centrality and body appreciation in diverse sexual orientation and gender identity groups

**DOI:** 10.1186/s12939-024-02268-3

**Published:** 2024-09-18

**Authors:** Nikola Komlenac, Kristina Stockburger, Jennifer Birke, Margarethe Hochleitner

**Affiliations:** Institute for Diversity in Medicine, Medical University of Innsbruck, Fritz-Pregl Strasse 3, Innsbruck, 6020 Austria

**Keywords:** Body appreciation, LGBTQ+, Social group, In-group pressure, Discrimination, Social cure model

## Abstract

**Background:**

The level of experienced sociocultural pressure to have an idealized body can vary depending on a person’s gender identity and sexual orientation. The current study explored whether differences in levels of body appreciation among people with different sexual orientations and gender identities vary because of differing levels of experienced pressure by in-group members and varying levels of experienced hostile behaviors because of their looks or body. Thereby, the study tests the social cure model, according to which high levels of identity centrality are associated with better mental health.

**Methods:**

An online cross-sectional questionnaire study was conducted with 1,587 people (51.3% cisgender women, 39.3% cisgender men, 9.5% non-binary; 52.9% identified as heterosexual, 27.7% identified as bisexual/pansexual, 11.2% identified as gay/lesbian, 8.2% identified as asexual/demi/queer; *M*_age_ = 32.9, *SD* = 12.6) from German-speaking countries. Variables were assessed with German-language versions of the Multidimensional and Multicomponent Measure of Social Identification, Body Appreciation Scale-2, the Perceived Stigmatization Questionnaire, and the Sociocultural Attitudes Towards Appearance Questionnaire-4, revised. A manifest-path model was calculated.

**Results:**

Non-binary persons reported lower levels of body appreciation than did cisgender men and sexual minority persons reported lower levels of body appreciation than did heterosexual persons. Furthermore, sexual minority persons experienced more hostile behaviors directed towards them because of their looks or body than did heterosexual persons. Similarly, non-binary persons experienced more hostile behaviors than did men. Non-binary persons were subjected to lower levels of in-group pressure than were men. Gay/lesbian persons and asexual persons were subjected to lower levels of in-group pressure than were heterosexual persons. More hostile behaviors and stronger in-group pressure were related to lower body appreciation. In cisgender women and men indirect links revealed associations between strong identity centrality and low levels of body appreciation through the mediator of high in-group pressure.

**Conclusions:**

Data in sexual minority individuals or non-binary persons supported the social cure model, according to which persons can find support and validation for their looks and body from in-group members. In cisgender women and men, strong identification as a woman or man can be related to stronger in-group pressure and in turn to lower body appreciation.

**Supplementary Information:**

The online version contains supplementary material available at 10.1186/s12939-024-02268-3.

## Introduction

Body satisfaction refers to a person’s thoughts, evaluations, and feelings about their body [1], whereas, body appreciation is characterized by holding favorable opinions, evaluations, or feelings toward one’s body [2]. However, a considerable proportion of the population (up to 46%) reports not being satisfied with at least one aspect of their body at some point in their life [3].

It is well-documented that gender identity is linked to body appreciation. Namely, it has been reported that on average men report higher levels of body appreciation than do women or persons with trans or nonbinary gender identities [4, 5]. Furthermore, sexual orientation has been found to be related to body satisfaction [6]. Namely, gay and bisexual men have lower levels of body satisfaction than heterosexual men [6]. On the contrary, lesbian or bisexual women often experience levels of body satisfaction similar to those of heterosexual women [6–8], or higher levels of body satisfaction [7, 9].

Sociocultural pressure to adhere to a certain body standard is one major factor related to body appreciation [10]. The current study used the social cure model [11] to investigate whether identity centrality (i.e., the degree to which a specific social identity is important to an individual) [12] with regard to a particular sexual orientation and gender is linked to body appreciation. The current study adds to the existing literature by considering whether identity centrality is linked to in-group members’ pressure to have muscular or thin bodies, experiences of hostile behaviors, and finally body appreciation. Therefore, we will analyze whether associations among these variables vary depending on a person’s sexual orientation and gender identity.

### Culturally held standards for the body, stigma, and pressure

Low body satisfaction is often rooted in the inability to create and recreate culturally held standards for the body [13–15]. In many Western countries, including Austria and Germany, such culturally held standards prescribe heterosexual and cisgender identities and often define unattainable body characteristics as ideal [1, 15]. In women such idealized body characteristics include thinness (and low weight), elegance, youth, slim but full-breasted figures, and firm-looking bodies [1]. In men, idealized bodies are often described as mesomorphic, which is characterized by the body having little body fat while being muscular [1]. Culturally held standards for the body often position signs of aging, including greying hair, wrinkles, sagging skin, and weight (gain), as unattractive. Additionally, having a healthy body is seen as a prerequisite for physical attractiveness [16–21]. Finally, bodies with disabilities are deemed to deviate from culturally held standards [22].

People are often pressured by others to adhere to body standards. A meta-analysis summarizes the link between higher levels of sociocultural pressure and lower levels of body appreciation [10]. Thereby, previous research shows that the level of experienced sociocultural pressure can vary depending on a person’s gender identity. One explanation for lower levels of body appreciation in women as compared to men, can be that women experience stronger sociocultural pressure to adhere to body standards than men [23–25]. However, studies about body appreciation that consider gender beyond binary categories such as studies on experienced pressure in non-binary persons are lacking in literature [5, 26]. The current study will add to the literature by including perspectives and experiences of non-binary, gender fluid, or genderqueer (whereby the umbrella term non-binary will be used henceforth) [27].

In contrast to non-binary persons transgender persons more often report not being satisfied with their bodies because of their genitalia and other sexually dimorphic body parts [28]. Many, transgender persons are likely to have wishes for or to undergo gender-affirming intervention [29]. Undergoing gender-affirming interventions is linked to better mental health in a large study among 28 European transgender persons. Especially in countries with low structural stigma (i.e., discriminatory laws, social policies, and public attitudes), gender-affirming interventions were linked to fewer symptoms of depression and stronger life satisfaction because of decreased identity concealment [30]. In the current study experiences of transgender persons will not be considered, because the consideration of gender-affirming interventions was beyond the scope of the current study.

Beyond a person’s gender identity, experiencing sociocultural pressure to adhere to body standards is also related to a person’s sexual orientation. Namely, gay men have been found to experience stronger pressure to adhere to body standards than heterosexual men [23]. Specifically, in comparison to heterosexual men, gay men have been found to be more concerned with having a thin body and less concerned with being muscular [8, 23]. Lesbian women, on the other hand, did not differ in experiencing pressure to adhere to body standards from heterosexual women [23]. Experienced pressure to adhere to body standards in bisexual, pansexual, or asexual persons has not been explored extensively.

Differences in body appreciation depending on a person’s gender identity and sexual orientation have been linked to other factors in addition to varying degrees of experienced pressure. It has been proposed that in comparison to heterosexual men, heterosexual women, gay men or bisexual women or men are more likely to be sexually objectified and to self-objectify themselves (i.e., are judged and judge themselves by considering only their bodies (as sexual objects) and whether those bodies adhere to body standards) [31–33]. In this regard, persons subjected to the “male gaze” (e.g., heterosexual women, gay men, bisexual/pansexual women or men), i.e., a sexualized gaze most often from men, are more likely to self-objectify themselves [34–36]. Non-binary people can be subject to sexual objectification, too. A qualitative study reports experiences of non-binary people being perceived and sexualized as women, or being sexualized by their body’s reproductive capacity. Among other consequences of being sexually objectified, non-binary persons reported not being satisfied with their bodies or modifying their appearance [37].

Finally, the frequency of experiencing hostile behaviors by others because of one’s looks or body can differ depending on a person’s gender identity and sexual orientation. For instance, gay, lesbian, bisexual, and asexual persons have been found to be more frequently subject to weight-related teasing than heterosexual persons [38]. Furthermore, gay men who strongly identify with a gay community or have greater gay community involvement were found to be more concerned about having a muscular body than gay men who did not identify with a gay community [39]. In a qualitative study, gay men report how the pressure is experienced to make them believe that having a body aligned with body standards is “mandatory” [40]. However, those findings that gay community identification is linked to greater concerns about muscularity are inconsistent across the literature [39].

### Social cure model

The social cure model suggests that social identities can be psychological resources and that greater social identification or community connectedness leads to greater levels of psychological well-being [11]. Community connectedness is defined as the convergence of individuals’ desire to belong to a larger collective, establish a mutually influential relationship with that collective, satisfy their individual needs and be rewarded through their collective affiliation, and construct a shared emotional connection [41]. Especially sexual and gender minority individuals might benefit from greater social identification or community connectedness because they often face minority stress, including stigmatization, prejudice, and discrimination, internalized negative judgments about themselves, or fear of discrimination due to their deviation from cis-heteronormativity. Central to the minority stress model is the link between frequent or intense minority stress and poor health. In line with the social cure model, the minority stress model includes community connectedness as a factor that can ameliorate the link between frequent or intense experiences of minority stress and poor health [42–44].

Studies supporting the social cure model report links between stronger LGBTQ+ (lesbian, gay, bisexual, transgender, queer, and other) community connectedness and lower levels of psychological distress or depressive symptoms in LGBTQ + individuals, especially in adolescents and emerging adults [45–47]. In a qualitative study gender and sexual minority individuals explain that their experience of community belonging improved their mental health [48]. In a questionnaire study among gender minority persons from the United States of America, stronger community connectedness was linked to fewer experiences of discrimination and lower levels of pressure to adhere to body standards of thinness [49].

Furthermore, the social cure model considers a person’s social identity and the degree to which the in-group identification is relevant to a person’s self-definition, i.e., identity centrality [50, 51]. Strong identity centrality is characterized by a person’s strong feeling of belonging, and their strong tendency to identify or describe themselves with a specific social identity [52]. Persons with strong identity centrality might perceive social support, such as positive identity-relevant affirmation [12], given by other social group members as being more benevolent than do persons with poor identity centrality [53]. So far, research has reported positive effects that indicate that higher levels of identity centrality are associated with fewer mental health symptoms, fewer physical health symptoms, or greater well-being. However, empirical findings are lacking about the links between identity centrality and body appreciation, especially in non-binary persons, or bisexual/pansexual and asexual persons [12].

## The current study

**Fig. 1:**
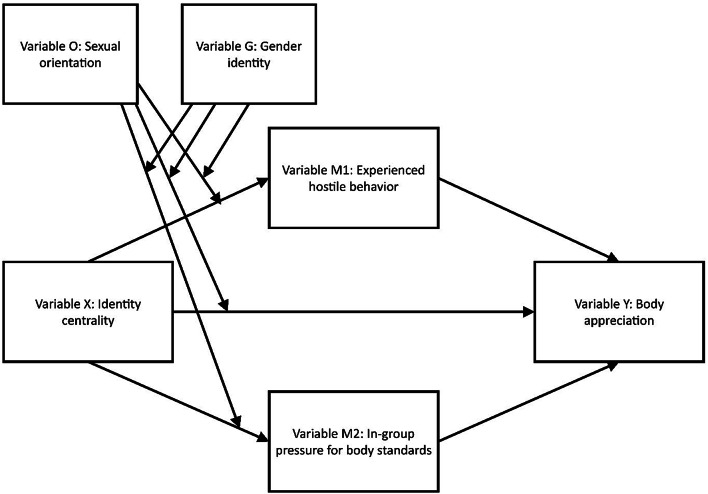
Manifest path model for testing the social cure model. *Note*: Body appreciation (variable Y) was predicted from a person’s level of identity centrality (variable X). Thereby, experienced hostile behavior (Mediator 1, variable M1) or/and levels of pressure exerted by persons in the in-group to have muscular or thin bodies (Mediator 2, variable M2) were tested as mediators. It was tested whether associations varied depending on a person’s sexual orientation (Moderator, variable O; heterosexual was set as reference) and/or gender identity (Moderator, variable G, man was set as reference)

The current study applies the social cure model [11] and analyzes whether a person’s identity centrality can be a psychological resource. Thus, it will be tested whether a person’s strong identity centrality is linked to infrequent experiences of pressure to adhere to body standards of thinness and muscularity, or infrequent experiences of hostile behaviors because of one’s body, and consequently to higher levels of body appreciation (Fig. 1).

In the current study, the group of heterosexual persons was set as the reference group when considering sexual orientation, and men were set as reference group when considering gender because heterosexual persons and/or men can be expected to experience lower levels of sociocultural pressure to adhere to body standards [23–25], be least frequently exposed to the male gaze [35, 36] or weight-related teasing [38] and can be expected to have the highest level of body appreciation [4, 5] in comparison to women and/or sexual and gender minority persons.

According to the social cure model [11], strong identity centrality might help legitimize and accept bodies that deviate from strict body standards, especially in persons with sexual and gender minority social identities. Thus, it can be hypothesized that strong identity centrality is linked to perceiving less pressure to strictly conform to body standards, to infrequent experiences of stigmatization (e.g., hostile behaviors), and consequently to higher levels of body appreciation (Fig. 1).

## Method

### Procedure

The medical university’s Ethics Committee confirmed that under Austrian law the current study did not require formal approval by an ethics committee [54, 55]. Recruitment of study participants started in December 2022 and ended in February 2023. The majority of the sample (56.6%) was recruited via the crowdsourcing service [56, 57] Prolific Academic (Prolific, London, UK). Participants recruited through Prolific Academic needed to be located in Germany, Austria, or Switzerland. To reach sexual and gender minority persons, 450 persons who reported identifying as LGBTQ + on Prolific Academic’s pre-selection questionnaires were recruited. Additionally, 150 women and 350 men who did not identify as LGBTQ + were recruited through Prolific Academic. Participants received GBP 3.0 to GBP 5.0 as compensation for their participation. The study was also promoted on Facebook and Instagram by using authors’ private accounts [58]. In this way, 35.9% of the participants were recruited for the study. Finally, all students at an Austrian medical university were invited by e-mail to participate in the study. The minority of participants (6.2%) were recruited via e-mail invitation (Table 1)^1^. Participants recruited through Facebook and Instagram or e-mail did not receive any compensation for their participation.

**Table 1:** Sociodemographic description of the sample

Variable		All *N* (%)	Women *N* (%)	Men *N* (%)	Non-binary persons *N* (%)
Whole sample		1587 (100)	814 (51.3)	623 (39.3)	150 (9.5)
Sexual Orientation (Identifying as…)					
	Heterosexual	820 (52.9)	381 (48.3)	431 (69.7)	8 (5.6)
	Gay/lesbian	429 (27.7)	278 (35.2)	79 (12.8)	72 (50.3)
	Bisexual/ pansexua	174 (11.2)	63 (8.0)	94 (15.2)	17 (11.9)
	Asexual/ demi/ queer	127 (8.2)	67 (8.5)	14 (2.3)	46 (32.2)
Relationship					
	Single	655 (41.3)	327 (40.2)	264 (42.4)	64 (42.7)
	Relationship without sexual activity	71 (4.5)	40 (4.9)	17 (2.7)	14 (9.3)
	In relationship	751 (47.3)	396 (48.6)	304 (48.8)	51 (34.0)
	Open/poly relationship	110 (6.9)	51 (6.3)	38 (6.1)	21 (14.0)
Nationality					
	German	1117 (70.4)	548 (67.3)	452 (72.6)	117 (78.0)
	Austrian	203 (12.8)	130 (16.0)	55 (8.8)	18 (12.0)
	Other	267 (16.8)	136 (16.7)	116 (18.6)	15 (10.0)
Education					
	Primary school & vocational training	196 (12.4)	73 (9.0)	97 (15.6)	26 (17.3)
	University entrance level	606 (38.2)	322 (39.6)	217 (34.8)	67 (44.7)
	University degree	785 (49.5)	419 (51.5)	309 (49.6)	57 (38.0)
Employment					
	Working	814 (51.3)	397 (48.8)	367 (58.9)	50 (33.3)
	Education	588 (37.1)	325 (39.9)	189 (30.3)	74 (49.3)
	Not in paid work	185 (11.7)	92 (11.3)	67 (10.8)	26 (17.3)
Recruited					
	E-Mail	98 (6.2)	64 (7.9)	32 (5.1)	2 (1.3)
	Facebook/Instagram	570 (35.9)	318 (39.1)	152 (24.4)	100 (66.7)
	Prolific	899 (56.6)	420 (51.6)	435 (69.8)	44 (29.3)
	Other	20 (1.3)	12 (1.5)	4 (0.6)	4 (2.7)

The study was hosted on SoSci: der onlineFragebogen (SoSci Survey GmbH, Munich, Germany). The study was described as being about social groups, belonging, body image, and well-being. Participation was voluntary and anonymous. All participants were able to withdraw from participation at any time. Participants were able to access the questionnaire only after agreeing that their anonymous data would be saved and used for research. Participants were able to complete the questionnaire and receive compensation even if they left some or all questions unanswered.

On average participants needed 17.6 min to respond to all study questions. In total 2,667 persons entered the online questionnaire. Of those, 854 participants were excluded from the study because they did not respond to or responded incorrectly to two instructed response items (“Please select the response ‘Always”) [59, 60]. Participants (*n* = 16) who gave obvious incorrect responses were excluded, such as participants who expressed discontent about the study’s content in free-text responses to the question about participants’ gender, e.g., “I hate gender” or “half-god.” Some participants reported an unrealistic body mass index (BMI) of zero or an extremely large BMI (e.g., 210.53, 508.32). To handle outliers a statistical rule of thumb was applied and persons with a BMI larger than two standard deviations from the sample’s mean (larger than 54.6) were excluded [61, 62]. Some participants (*n* = 86) could not be considered because they did not respond to the main variables that were included in the analyses. Finally, 113 persons who identified as transgender (or did not indicate their gender) were excluded from the study because the consideration of gender-affirming interventions or gender dysphoria [29] was beyond the scope of the current study.

### Measures

#### Sociodemographic information

Sociodemographic information was assessed with self-constructed questions about participants’ gender identity [63], sexual orientation (i.e., identity label) [64], relationship status, highest level of education, and nationality (Table 1). For each question, participants could choose from several response options and give a free-text response. For analysis, many free-text responses (e.g., “committed open relationship with sexual activity”) were categorized into predefined response options (e.g., “open/poly-relationship”). Participants reported their age (years), height (meters), and weight (kilogram) with free-text responses. For analysis, the body mass index (BMI) was calculated as weight in kilograms divided by height in meters squared [65].

#### Identity centrality

In order to measure how important social identity is for a person’s self-concept, i.e., whether social identity plays a central aspect in a person’s sense of who they are, the centrality scale of the Multidimensional and Multicomponent Measure of Social Identification was used [50]. For the current study, the German version was used [66]. Thereby, identity centrality was measured with three statements, to which participants could indicate their level of agreement on a five-point Likert scale (1 = totally disagree, 5 = totally agree; example item, “I often think about the fact that I am [in-group]”).

Participants could choose from a list (of sexual orientation identity labels, gender identity labels, the intersection of sexual orientation identity labels and gender identity labels, or less specific social group labels, such as “sexual and gender minority” [63, 64]; Supplemental Material S2) the social group participants referred to when indicating their identity centrality with one item that has was used in previous studies [52, 67]. Thereby, participants were informed that all people can refer to and identify with social groups (e.g., women, men, students, Germans, Europeans, Catholics). Participants were instructed to choose from a list of 27 different sexual and gender groups (Supplemental Material S1) the group they most strongly felt they belonged to, most likely identified with, or described themselves as [52].

Items of Multidimensional and Multicomponent Measure of Social Identification [50] were modified so that “[in-group]” was replaced with the social identity that participants had indicated (Supplemental Material S1). A mean score across the three items was calculated and, thus, higher scores indicated higher levels of identity centrality with one’s own social identity. The original internal consistency of the scale was α = 0.80 – 0.87 (referring to being Dutch, European, or a member of the University of Amsterdam) [50]. The previously validated German version included gender identification (as man or woman) and had an internal consistency of α = 0.67 [66]. In the current study, all Cronbach’s alphas were larger than 0.78 [68; Tables 2 and 3].

**Table 2:** Descriptive statistics by gender

Variables	All		Gender						F(2, 1587)	η^2^
			Women		Men		Non-binary			
	M (SD)	α	M (SD)	α	M (SD)	α	M (SD)	α		
Age	32.9 (12.6)		33.9 (12.4)		32.9 (13.1)		29.0^†^ (9.4)		9.4***	0.01
BMI	25.4 (6.0)		25.9^†^ (5.3)		25.0 (6.3)		25.6 (7.1)		4.5*	0.01
Hostile behavior^a^	1.5 (0.7)	0.91	1.5 (0.6)	0.90	1.5 (0.6)	0.91	1.8^†^ (0.8)	0.91	17.4***	0.02
Pressure in-group^b^	2.1 (0.9)	0.83	2.1 (0.9)	0.85	2.1 (1.0)	0.83	1.9^†^ (0.8)	0.78	5.6**	0.01
Identity centrality^b^	3.3 (1.1)	0.84	3.0^†^ (1.1)	82	3.4 (1.0)	0.82	4.0^†^ (0.9)	0.83	65.7***	0.08
Body appreciation^c^	3.4 (0.8)	0.94	3.4 (0.8)	0.94	3.4 (0.8)	0.93	3.1^†^ (0.8)	0.94	14.6***	0.02

*Note*^a^Possible range: 1 (never) – 5 (always); ^b^Possible range: 1 (totally disagree) – 5 (totally agree); ^c^Possible range: 1 (never) – 5 (always [appreciate my body])

^†^Significant contrast between men and people with a different gender

BMI = body mass index; α = Cronbach’s alpha

* *p* < .05; ** *p* < .01; *** *p* < .001

**Table 3:** Descriptive statistics by sexual orientation

Variables	Sexual orientation								F(3, 1550)	η^2^
	Heterosexual		Gay/lesbian		Bisexual/ pansexual		Asexual/ demi/ queer			
	M (SD)	α	M (SD)	α	M (SD)	α	M (SD)	α		
Age	35.7 (13.3)		28.9^†^ (9.7)		33.2^†^ (12.9)		28.4^†^ (10.8)		32.7***	0.06
BMI	25.4 (5.6)		25.8 (6.6)		24.6 (5.6)		25.0 (6.8)		1.5	0.00
Hostile behavior^a^	1.5 (0.6)^1^	0.90	1.7^†^ (0.7)	0.91	1.7^†^ (0.7)^3^	0.91	1.6^†^ (0.7)	0.91	10.2***	0.02
Pressure in-group^b^	2.2 (0.9)	0.83	2.0^†^ (0.9)	0.82	2.3^†^ (1.0)	0.84	1.7^†^ (0.8)	0.83	19.2***	0.04
Identity centrality^b^	3.1 (1.1)^2^	0.81	3.5^†^ (1.1)	0.86	3.8^†^ (0.9)^4^	0.78	3.7^†^ (1.0)	0.83	31.0***	0.06
Body appreciation^c^	3.5 (0.8)	0.94	3.3^†^ (0.8)	0.94	3.3^†^ (0.8)	0.94	3.1^†^ (0.8)	0.93	12.9***	0.02

*Note*^a^Possible range: 1 (never) – 5 (always); ^b^Possible range: 1 (totally disagree) – 5 (totally agree); ^c^Possible range: 1 (never) – 5 (always [appreciate my body])

^†^Significant contrast between heterosexual persons and persons with another sexual orientation

^1^Range of values in heterosexual non-binary persons (*n* = 8): 1.0–3.2

^2^Range of values in heterosexual non-binary persons (*n* = 8): 2.0–4.0

^3^Range of values in bisexual/pansexual non-binary persons (*n* = 17): 1.0–2.8

^4^Range of values in bisexual/pansexual non-binary persons (*n* = 17): 3.0–5.0

BMI = body mass index; α = Cronbach’s alpha

*** *p* < .001

#### Body appreciation

The level to which participants held favorable opinions toward and respected their body, i.e., level of body appreciation, was assessed with the Body Appreciation Scale-2 (BAS-2) [2]. This questionnaire consists of ten statements about respecting, appreciating, or holding favorable opinions toward the body (e.g., “I feel good about my body”), to which participants indicated on a five-point Likert scale how often they thought about their body in such a manner (1 = never, 5 = always). Mean scores across the ten items were calculated, whereby higher mean scores indicate higher levels of body appreciation. For the current study, the German version of the BAS-2 was used [69]. Initially, the questionnaire was developed in women with an internal consistency of α = 0.93 – 0.94. The German version of the BAS-2 was validated in women and men. The previous validation study confirmed that the scale had strong internal consistency (ω = 0.93 – 0.95) and was invariant across women and men [69]. Other studies confirmed that the BAS-2 is invariant across women and men [5, 70] and across heterosexual and sexual minority people [70, 71]. In the current study all internal consistencies were above 0.93 (Table 2).

#### Pressure to adhere to body standards

Participants were asked whether members of their in-group pressured them to have muscular or thin bodies (e.g., “My peers encourage me to get thinner”; 4 items) with the Sociocultural Attitudes Towards Appearance Questionnaire-4, revised (SATAQ-4R) [72]. Items that previously referred to “peers” were modified so that the items referred to the participants’ social group. All items were in the German language [73]. Participants indicated their agreement with statements on a five-point Likert scale (1 = totally disagree, 5 = totally agree). A higher mean score on a scale indicated higher levels of pressure exerted by persons of the social in-group to have muscular or thin bodies. The internal consistencies of the scales were reported with α = 0.82 – 0.92 in women and α = 0.75 – 0.91 in men [72]. Furthermore, a previous study reported measurement invariance across gender (cisgender women vs. cisgender men) and sexual orientation of the SATAQ-4 [74]. In the current study, internal consistencies (Cronbach’s alphas) were larger than 0.82 (Tables 2 and 3).

#### Experiencing hostile behaviors because of one’s body

With the hostile behavior subscale of the Perceived Stigmatization Questionnaire (PSQ) [75] participants were asked how often (1 = never, 5 = always) they experienced hostile behavior (e.g., “People pick on me”) because of their looks or body. For the current study, German-language items were used [76]. The mean score across the five items of the scale was calculated, whereby higher mean scores indicated that participants often experience hostile behavior towards them because of their looks or body. Originally, the scale was developed and used in burn survivors [75] and was reported to have internal consistencies of 0.73 [76; German-language] – 0.89 [75; original]. In the current study all internal consistencies (Cronbach’s alphas) were larger than 0.91 (Tables 2 and 3).

### Statistical analysis

Descriptive statistics (means, standard deviations, percentages) as well as correlations between continuous variables are reported. Variables did not markedly violate the assumption of normal distribution (skew: -0.4–1.3; kurtosis: -0.9–2.1) [77]. Differences in mean scores between people with different gender identities and sexual orientations were calculated with ANOVAs, whereby men were set as the reference group when considering gender, and heterosexual people were set as the reference group when considering sexual orientation for planned contrasts [78].

To test the current study’s research question a manifest-path model was calculated. In the model body appreciation (variable Y) was predicted from a person’s level of identity centrality (variable X) (Fig. 1). The model tested whether the link between body appreciation and identity centrality was mediated by the level of experienced hostile behavior (Mediator 1, variable M1) or/and levels of pressure exerted by persons in the in-group to have muscular or thin bodies (Mediator 2, variable M2). Furthermore, it was tested whether the link between body appreciation and identity centrality varied depending on a person’s sexual orientation (Moderator, variable O; heterosexual was set as reference) and/or gender identity (Moderator, variable G, man was set as reference). Age, relationship status, BMI, nationality, education, and employment were considered co-variables. The BMI was considered as co-variable because of known links between the BMI and levels of body appreciation and recommendations to control for body size when researching body appreciation [79]. The model was calculated with the PROCESS macro version 4.1 [80] for SPSS, version 29.0 (IBM Corp., Armonk, NY, USA). Direct as well as indirect links between variable X and variable Y were calculated with the help of bootstrap bias-corrected 95% confidence intervals (bootstrap sample was *n* = 5000). Significant results were indicated when *p* ≤ .05 or when 95% confidence intervals did not include zero [80].

Based on a study that applied the intragroup status and health model and tested links between identity centrality, perceived discrimination and mental health, medium effect sizes (β > 0.26) were expected [81]. To detect medium effect sizes a sample of 148 participants is recommended (β > 0.26, α = 0.05, power = 0.8) [82, 83]. The current study exceeds the suggested minimum sample size. Furthermore, the currently used statistical method is known to be reliable and robust also in small samples [80, 84].

## Results

### Participants

The sociodemographic characteristics of the sample are reported in Table 1. Overall, 1,587 persons (51.3% cisgender women, 39.3% cisgender men, and 9.5% non-binary persons) participated in the study. Participants were on average 32.9 (Table 2; range: 18–76) years old. The BMI of participants ranged from 14.5 to 54.6 and averaged 25.4 (Table 2). The majority of participants were from Germany, with other participants being from Austria or a different country (Table 1). Around half of the participants were in a relationship. Other participants reported being single, being in an open or poly-relationship, or being in a relationship without partnered sexual activity (Table 1). Nearly half of the participants had a university degree and were working in paid work (Table 1).

### Descriptive statistics

Overall, participants reported moderate levels of identity centrality (Table 2). Cisgender women reported lower levels of identity centrality, whereas non-binary persons reported higher levels of identity centrality than did cisgender men. Compared to heterosexual persons gay/lesbian, bisexual/pansexual, or asexual persons had higher levels of identity centrality (Table 3). An overview of the social group participants referred to when indicating their identity centrality can be found in Supplementary Materials (S2). Thereby around 17% of participants identified women, 11% men, 11% heterosexual men, and 9% queer persons as the social group they most strongly felt they belonged to, most likely identified with, or described themselves as.

On average, participants reported rarely experiencing hostile behavior towards themselves because of their bodies or looks (Table 2). In addition, non-binary persons reported more frequently experiencing hostile behaviors than did cisgender men (Table 2) and sexual minority persons reported more frequently experiencing hostile behaviors than did heterosexual persons (Table 3).

Across the whole sample, participants reported that it was unlikely that they would experience pressure from in-group members to adhere to heteronormative body standards. In comparison to cisgender men, non-binary persons experienced lower levels of pressure from in-group members to adhere to cis-normative body standards (Table 2). People who identified as heterosexual reported more frequently experiencing being pressured by in-group members to adhere to body standards than did people who identified as gay/lesbian or asexual (Table 3). Pansexual/bisexual persons reported to be pressured to adhere to heteronormative body standards more often than heterosexual persons.

Participants reported having moderate levels of body appreciation. Non-binary persons reported lower levels of body appreciation than did cisgender men (Table 2). Sexual minority persons had lower levels of body appreciation than heterosexual persons (Table 3).

Correlations between variables are presented in Table 4. Frequent experiences of hostile behavior were weakly [85] linked to higher levels of identity centrality, and in-group pressure to adhere to body standards. Higher levels of body appreciation were moderately [85] linked to lower levels of experienced hostile behaviors, and weakly linked to higher levels of identity centrality, and lower levels of in-group pressure to adhere to body standards (Table 4).

**Table 4:** Correlations between variables

Variable	6.	7.	8.	9.	10.	11.	12.	13.	14.	15.
1. Cis men vs. cis women	− 0.01	0.20***	− 0.01	− 0.04	0.01	− 0.08**	0.02	0.06*	0.08**	− 0.01
2. Cis men vs. non-binary	0.19***	0.35***	− 0.12***	− 0.16***	0.00	− 0.02	− 0.07*	− 0.07*	0.18***	− 0.18***
3. Heterosexual vs. gay/lesbian	0.13***	0.15***	− 0.11***	− 0.25***	0.04	0.03	− 0.03	− 0.05	0.08**	− 0.12***
4. Heterosexual vs. bi/pansexual	0.13***	0.26***	0.07*	− 0.07*	− 0.12***	− 0.06	− 0.04	0.01	0.06	− 0.10**
5. Heterosexual vs. asexual	0.09**	0.16***	− 0.19***	− 0.17***	− 0.27***	− 0.02	− 0.11***	− 0.11***	0.14***	− 0.16***
6. Hostile behavior		0.07**	0.25***	− 0.03	− 0.09***	0.21***	− 0.01	− 0.10***	0.08**	− 0.33***
7. Identity centrality			0.08***	− 0.02	− 0.01	− 0.03	0.04	0.02	0.04	0.05*
8. Pressure in-group				− 0.01	0.01	0.13***	0.05	0.08**	0.00	− 0.19***
9. Age					0.08**	0.29***	− 0.08***	0.14***	− 0.12***	0.07**
10. Relationship status						− 0.02	0.03	0.15***	− 0.14***	0.15***
11. BMI							− 0.06*	− 0.02	− 0.02	− 0.22***
12. Nationality								0.08**	0.01	0.05
13. Education									− 0.25***	0.11***
14. Employment										− 0.10***
15. Body appreciation										

*Note* BMI = body mass index

*p < .05, **p < .01, ***p < .001

### Manifest path model

#### Mediator 1: Hostile behaviors

In the manifest path model, a significant three-way interaction indicated that identity centrality was linked to experiencing hostile behavior towards themselves because of their body or looks depending on the person’s identity centrality, sexual orientation, and gender (Table 5). In heterosexual men high levels of identity centrality were linked to more frequent experiences of hostile behaviors (*b* = 0.07, *SE* = 0.03, *p* = .038), whereas in gay men identity centrality was not linked to experiences of hostile behaviors (*b* = 0.01, *SE* = 0.08, *p* = .884; results did not support the social cure model). In non-binary persons, identity centrality was linked to less frequent experiences of hostile behaviors when persons identified as heterosexual (*b* = -0.75, *SE* = 0.34, *p* = .025) or bisexual/pansexual (*b* = -0.64, *SE* = 0.30, *p* = .036; results supported the social cure model), whereby non-binary persons who identified as gay/lesbian experienced more frequent hostile behaviors the higher the level of their identity centrality was (*b* = 0.24, *SE* = 0.10, *p* = .020; results did not support the social cure model). In persons with another sexual orientation and/or gender identity centrality was not linked to experiences of hostile behaviors (all |*b*s| < 0.37, *p*s ≥ 0.057; results did not support the social cure model).

**Table 5:** Model path coefficients showing links between identity centrality and experiencing hostile behaviors because of one’s body

Predictor	B	SE B	95% CI for B	
			LL	UL
Identity centrality	-0.05	0.05	-0.15	0.04
O1: Heterosexual vs. gay/lesbian	-0.03	0.06	-0.15	0.09
O2: Heterosexual vs. bi/pansexual	0.22*	0.11	0.01	0.43
O3: Heterosexual vs. asexual	-0.06	0.09	-0.23	0.12
Identity centrality x O1	0.15**	0.06	0.04	0.26
Identity centrality x O3	0.18*	0.08	0.02	0.34
G1: Cis men vs. cis women	-0.07	0.06	-0.18	0.04
G2: Cis men vs. non-binary	0.29**	0.09	0.11	0.46
Identity centrality x G2	-0.23**	0.09	-0.39	-0.06
O3 x G2	-0.29*	0.14	-0.56	-0.03
Identity centrality x O1 x G2	0.36***	0.10	0.17	0.56
Age	-0.01**	0.00	-0.01	0.00
Relationship status	-0.05*	0.02	-0.09	-0.01
BMI	0.01***	0.00	0.00	0.02
Nationality	0.02	0.03	-0.03	0.08
Education	-0.05	0.03	-0.11	0.01
Employment	0.01	0.03	-0.05	0.07

*Note* BMI = body mass index; CI = confidence; All other interactions *p* > .05; Model: *F*(29,1520) = 5.17, *p* < .001, *R*^2^ = 0.09

*p < .05, **p < .01, ***p < .001

The significant interaction between Gender x Sexual orientation (O3 x G2; Table 5) indicates that in persons who identify as heterosexual non-binary people experience more frequent hostile behaviors than men do, whereby this difference is smaller in asexual non-binary persons (Supplemental Material S3).

#### Mediator 2: In-group pressure

The significant interaction Identity centrality x Gender (Table 6) indicates that the association between identity centrality and in-group pressure is different in women and men. Namely, at low levels of identity centrality, women experienced less in-group pressure to adhere to body standards than men, whereas at high levels of identity centrality women experienced similar levels of in-group pressure to adhere to body standards as did cisgender men (Supplemental Material S3; results did not support the social cure model).

**Table 6:** Model path coefficients showing links between identity centrality and experiencing pressure to adhere to body standards

Predictor	B	SE B	95% CI for B	
			LL	UL
Identity centrality	-0.01	0.17	-0.12	0.09
O1: Heterosexual vs. gay/lesbian	-0.07	0.05	-0.21	0.07
O2: Heterosexual vs. bi/pansexual	0.16	0.07	-0.07	0.40
O3: Heterosexual vs. asexual	-0.26**	0.12	-0.46	-0.06
G1: Cis men vs. cis women	-0.11	0.06	-0.23	0.02
G2: Cis men vs. non-binary	-0.07	0.10	-0.27	0.13
Identity centrality x G1	0.12*	0.06	0.00	0.24
O2 x G1	-0.28*	0.13	-0.55	-0.02
Age	-0.01***	0.00	-0.02	-0.01
Relationship status	0.00	0.02	-0.04	0.05
BMI	0.03***	0.00	0.02	0.04
Nationality	0.00	0.03	-0.06	0.06
Education	0.10**	0.03	0.03	0.17
Employment	0.02	0.03	-0.05	0.09

*Note* BMI = body mass index; CI = confidence; All other interactions *p* > .05; Model: *F*(29,1520) = 8.29, *p* < .001, *R*^2^ = 0.14

*p < .05, **p < .01, ***p < .001

Another significant interaction, namely Gender x Sexual orientation (O2 x G1; Table 6), indicated that in men, those who identify as heterosexual experience lower levels of in-group pressure to adhere to body standards than those men who identify as bi/pansexual (Supplemental Material S3).

#### Body appreciation

Experiencing frequent hostile behaviors and high levels of in-group pressure to adhere to body standards was linked to lower levels of body appreciation (Table 7). On the other hand, identity centrality was linked to higher levels of body appreciation.

**Table 7:** Model path coefficients showing links between identity centrality, hostile behaviors because of one’s body, experiencing pressure to adhere to body standards and body appreciation

Predictor	B	SE B	95% CI for B	
			LL	UL
Identity centrality	0.19***	0.04	0.10	0.27
Hostile Behaviors Because of One’s Body	-0.16***	0.02	-0.20	-0.11
Pressure to Adhere to Body Standards	-0.14***	0.02	-0.18	-0.09
O1: Heterosexual vs. gay/lesbian	-0.04	0.06	-0.15	0.08
O2: Heterosexual vs. bi/pansexual	-0.10	0.10	-0.30	0.09
O3: Heterosexual vs. asexual	-0.08	0.08	-0.24	0.08
Identity centrality x O1	-0.17**	0.05	-0.28	-0.07
G1: Cis men vs. cis women	0.04	0.05	-0.06	0.14
G2: Cis men vs. non-binary	-0.16	0.08	-0.32	0.00
Identity centrality x G1	-0.13**	0.05	-0.22	-0.03
O3 x G2	-0.28*	0.13	-0.53	-0.03
Age	0.00	0.00	0.00	0.01
Relationship status	0.07***	0.02	0.04	0.11
BMI	-0.03***	0.00	-0.03	-0.02
Nationality	0.02	0.02	-0.02	0.07
Education	0.07*	0.03	0.01	0.12
Employment	-0.04	0.03	-0.10	0.01

*Note* BMI = body mass index; CI = confidence; All other interactions *p* > .05; Model: *F*(31,1518) = 11.81, *p* < .001, *R*^2^ = 0.19

*p < .05, **p < .01, ***p < .001

The significant interaction term Identity centrality x Sexual orientation (Table 7) revealed that the link between identity centrality and body appreciation varied depending on sexual orientation. In heterosexual individuals (heterosexual men: *b* = 0.10, *SE* = 0.02, *p* = .003, heterosexual women: *b* = 0.12, *SE* = 0.04, *p* = .001, heterosexual non-binary persons: *b* = 0.59, *SE* = 0.31, *p* = .057; results supported the social cure model) stronger identity centrality was linked to higher levels of body appreciation. In gay/lesbian persons (gay men: *b* = 0. 07, *SE* = 0.08, *p* = .342, lesbian women: *b* = 0.01, *SE* = 0.04, *p* = .801, gay/lesbian non-binary persons: *b* = -0.05, *SE* = 0.09, *p* = .630) the link between identity centrality and body appreciation was not significant.

Furthermore, the interaction term Identity centrality x Gender (Table 7) revealed that the link between identity centrality and body appreciation varied depending on sexual orientation. The link between stronger identity centrality and higher levels of body appreciation was most prevalent in men (bi/pansexual men: *b* = 0. 18, *SE* = 0.09, *p* = .042, bi/pansexual women: *b* = -0. 03, *SE* = 0.10, *p* = .800; asexual men: *b* = 0. 59, *SE* = 0.18, *p* = .001, asexual women: *b* = 0. 14, *SE* = 0.09, *p* = .121; results supported the social cure model). Finally, based on the interaction Gender x Sexual orientation (O3 x G2; Table 7) it was revealed that the difference in body appreciation between heterosexual and asexual persons was larger in non-binary persons than in men (Supplementary Material S3).

The analysis of indirect links between identity centrality and body appreciation via the mediator hostile behaviors because of one’s body (Table 8) revealed that in heterosexual cisgender men and non-binary gay/lesbian persons stronger identity centrality was linked to lower levels of body appreciation (results did not support the social cure model). In non-binary heterosexual persons indirect associations revealed that strong identity centrality was linked to higher levels of body appreciation (results supported the social cure model).

**Table 8:** Indirect link between identity centrality and body appreciation via the mediator hostile behaviors because of one’s body

Sexual Orientation	Gender	*B*	Boot *SE B*	95% CI for *B*	
				*LL*	*UL*
Heterosexual	Cisgender man	-0.011*	0.006	-0.025	0.000
	Cisgender woman	-0.002	0.006	-0.014	0.009
	Non-binary person	0.117*	0.088	0.003	0.296
Gay/lesbian	Cisgender man	-0.002	0.018	-0.036	0.034
	Cisgender woman	-0.007	0.008	-0.023	0.007
	Non-binary person	-0.037*	0.018	-0.074	-0.004
Bisexual/pansexual	Cisgender man	-0.022	0.015	-0.054	0.006
	Cisgender woman	0.024	0.019	-0.012	0.062
	Non-binary person	0.099	0.067	-0.026	0.234
Asexual	Cisgender man	-0.057	0.031	-0.126	0.000
	Cisgender woman	0.003	0.013	-0.022	0.030
	Non-binary person	-0.006	0.020	-0.045	0.037

*Note* *significant effect

Finally, the analysis of indirect links between identity centrality and body appreciation via the mediator in-group pressure to adhere to body standards (Table 9) revealed an indirect link between high levels of identity centrality and low levels of body appreciation in heterosexual cisgender women and men (results did not support the social cure model).

**Table 9:** Indirect link between identity centrality and body appreciation via the mediator in-group pressure to adhere to body standards

Sexual Orientation	Gender	*B*	Boot *SE B*	95% CI for *B*	
				*LL*	*UL*
Heterosexual	Cisgender man	-0.021*	0.006	-0.034	-0.009
	Cisgender woman	-0.015*	0.007	-0.030	-0.001
	Non-binary person	0.063	0.067	-0.059	0.174
Gay/lesbian	Cisgender man	0.000	0.017	-0.035	0.034
	Cisgender woman	-0.014	0.008	-0.031	0.001
	Non-binary person	0.004	0.013	-0.021	0.030
Bisexual/pansexual	Cisgender man	-0.007	0.018	-0.045	0.030
	Cisgender woman	-0.017	0.019	-0.055	0.019
	Non-binary person	-0.045	0.052	-0.161	0.035
Asexual	Cisgender man	0.043	0.035	-0.034	0.110
	Cisgender woman	-0.012	0.014	-0.041	0.017
	Non-binary person	0.042	0.034	-0.019	0.112

*Note* *significant effect

## Discussion

The current study revealed that in comparison to cisgender men non-binary persons reported lower levels of body appreciation and gay/lesbian, bisexual/pansexual, or asexual persons reported lower levels of body appreciation than did heterosexual persons. The difference in body appreciation between heterosexual and asexual persons was larger in non-binary persons than in men.

Even though direct links between higher levels of identity centrality and higher levels of body appreciation were found in heterosexual men, indirect associations revealed that higher levels of identity centrality were linked to lower levels of body appreciation via higher levels of in-group pressure in heterosexual cisgender women and men. Furthermore, in heterosexual cisgender men and non-binary gay/lesbian persons stronger identity centrality was linked to lower levels of body appreciation via more frequent hostile behaviors because of one’s body. In non-binary heterosexual persons, indirect links between stronger identity centrality and higher levels of body appreciation because of the reduced frequency of hostile behaviors were found. In addition to the current study’s findings further research that uses an intersectional approach [86] might be fruitful when investigating the experiences of non-binary persons because non-binary gay/lesbian persons might experience multiple forms of oppression/discrimination because of deviating from multiple expectations of cis-heteronormativity (i.e., gender and sexual orientation) and thus experience more or different kinds of discrimination [87].

### Gender identity, sexual orientation and body appreciation

Consistent with previous findings, the current study found gay/lesbian, bisexual/pansexual, or asexual (sexual minority) persons reported lower levels of body appreciation than did heterosexual persons [6]. The current study adds findings about non-binary people having lower levels of body appreciation than cisgender men to the literature [5, 26]. Lower levels of body appreciation in non-binary people might result from non-binary persons’ dissatisfaction with sexually dimorphic body parts [29], which are often used to sexualize them. Among other consequences, non-binary persons reported modifying their appearance [37].

In the current study sexual minority persons and non-binary people reported experiencing more hostile behaviors directed towards them because of their looks or body than did heterosexual persons or men. In turn, experiencing more frequent hostile behaviors was linked to lower levels of body appreciation, as can be expected according to the minority stress model, which describes that minority stress (i.e., stigmatization, prejudice, and discrimination) can lead to poor mental health [43, 44, 88]. The current study adds to the scarce literature about non-binary person’s experiences of hostility directed towards them because of their looks or body that might result because of not conforming to cis-heteronormative body standards [5, 26, 89]. A past qualitative study suggests that non-binary persons might experience hostility that specific to their non-binary identity [37].

The current study supports previous suggestions of the need to consider minority stress and the ways affected persons cope with minority stress when helping sexual and gender minority people with poor body appreciation in clinical practice [6]. For example, important strategies include working out with sexual and gender minority persons effective and healthy coping strategies and how to cope with hostile behaviors [90]. Those recommendations also apply when addressing poor body appreciation in non-binary persons. When working with non-binary persons additional care should be put into avoiding cissexist behavior and into validating a non-binary identity [37].

The minority stress model and qualitative findings in non-binary persons [6, 37] also highlight that validation and support from the community can help sexual minority persons or non-binary persons cope after experiencing hostile behaviors. Feelings of value or acceptance for one’s gender minority identity have been linked to higher levels of body appreciation [91]. In the current study, non-binary persons reported experiencing lower levels of in-group pressure to adhere to body standards than did men. Similarly, gay/lesbian persons and asexual persons were subjected to lower levels of in-group pressure than heterosexual persons. Thus, social support indicated by low levels of in-group pressure might be beneficial for having higher levels of body appreciation, as the social cure model suggests [11].

However, the experience of relatively low levels of in-group pressure to adhere to body standards is not universal to all sexual minority groups. In the current study, bisexual/pansexual cisgender men experienced higher levels of in-group pressure to adhere to body standards than did heterosexual men. Bisexual/pansexual persons might face pressure and negative judgments because of violating norms of cis-heteronormativity in heterosexual or sexual minority communities. In sexual minority communities, some bisexual/pansexual persons might be confronted with dismissive attitudes and behaviors because of the presumption that bisexual/pansexual persons could easily “pass” as straight [92]. Bisexual/pansexual persons might also be pressured to behave in alignment with “binormative” stereotypes, i.e., a set of expectations about how bisexual/pansexual persons are or behave, such as plurisexual interest, dating same-gender partner(s), or enacting sociosexuality [92–95]. Thus, bisexual/pansexual cisgender men might experience lower levels of social support and feel more pressured to adhere to cis-heteronormative body standards [96, 97].

### Identity centrality and body appreciation

The direct link between stronger identity centrality and higher levels of body appreciation was most evident in men and heterosexual women. Heterosexual women with high levels of identity centrality might be more concerned about adhering to femininity ideologies that include cis-heteronormativity [98]. Women who strongly adhere to femininity ideologies have a stronger tendency to self-stereotype, i.e., perceive themselves as having characteristics that are associated with their in-group. In the past, higher levels of self-stereotyping in women were linked to higher levels of body appreciation [99], which is consistent with the current study’s findings. Similarly, men who appreciate their bodies might feel their masculinity validated by having a body that complies with body standards [100] and thus have higher levels of identity centrality.

The current study’s findings in cisgender men and cisgender heterosexual women did not support the social cure model [11] because indirect links that were small in effect size [85] revealed associations between strong identity centrality and low levels of body appreciation because of high in-group pressure in heterosexual cisgender women and men. Additionally, strong identity centrality and low levels of body appreciation were associated in heterosexual cisgender men because of frequent experiences of hostile behavior. Thus, especially in cisgender men having cis-heteronormative bodies might be related to social status [101, 102]. Therefore cisgender men with strong identity centrality might experience higher levels of pressure to adhere to body standards and experience devaluation when not fulfilling body standards [81]. As indirect effects were small [85], indirect links should be interpreted with caution.

### Implications

On the one hand, the current study supports interventions against minority stress [43, 44] that focus on a sexual and gender minority person’s community connection [103]. Increasing and helping build community connections can be of help in finding acceptance and interpersonal support, and thus be an intervention against minority stress [48, 104].

However, especially in heterosexual cisgender men identity centrality might be linked to higher levels of pressure to adhere to body standards and the experience of devaluations when not fulfilling body standards, in part because of social status being related to body appearance [101, 102]. Furthermore, men’s perception of their masculinity might be influenced by having bodies that comply with body standards [100]. Therefore, interventions that target men’s low body satisfaction need to consider men’s body’s potential influence on social status [101, 102] and ways by which men construct masculinity and their feeling of being masculine [100, 105]. A person’s self-ascribed masculinities (i.e., the degree to which masculinity is important for a person’s sense of self-worth) need to be explored. Additionally, situational cues and meanings of masculinity, ways to enact masculinity, and the “costs” and benefits of enacting “alternative” masculinities (i.e., masculinities not associated with the highest social status) can be addressed during interventions targeting men’s low body satisfaction [106, 107].

Finally, the current study highlights that people with similar sexual orientation or gender identity might occupy different social locations (i.e., bi/pansexual persons having different experiences from other sexual minority persons) [86]. Therefore, studies that investigate health disparities of sexual and gender minority individuals [e.g., 108, 109] should include a measure of the persons’ social identity [110] and identity centrality.

### Limitations

The study’s proportion of people with a tertiary education degree was larger than the proportion of people with a university degree in the German [111] or Austrian population [112]. Furthermore, up to one-third of the sample was pursuing an education at the time of study participation [113]. Studies that include only university students or people with a university degree often find associations with larger effect sizes than do studies with samples more representative of the population [114]. Additionally, the results (and the literature review) of the present study rely on data from samples of Western, Educated, Industrialized, Rich, andDemocratic samples [113]. Thus, the current study is not representative of multicultural diversity, and findings and conclusions might not be generalizable to non-Western societies, (e.g., Asia, Africa).

The study used a relatively large sample. Nevertheless, the number of individuals who identified as gay/lesbian, asexual, and/or non-binary was relatively small [82]. Thus, some associations that exist might not have been revealed in gay/lesbian, asexual, and/or non-binary individuals. Furthermore, the bootstrapped confidence intervals can be inaccurate (inflatethe Type I error), especially in small samples [115]. To increase confidence in the present study’s findings, future research needs to apply more sophisticated or extensive recruitment methods to increase the number of sexual and gender minority persons.

Even though some of the currently used questionnaires were validated in sexual minority individuals (BAS-2 [70, 71]; SATAQ-4R [74]), instruments were not validated for non-binary individuals. Nevertheless, all internal consistencies of instruments were acceptable [68].

The cross-sectional design of the current study does not allow any conclusions to be drawn about the causality or directionality of effects. For instance, high levels of body appreciation in cisgender heterosexual women or men with high levels of identity centrality might be the result of those persons’ perception that their bodies meet body standards for women [1]. Thus, the perception of having bodies that meet standards might result in persons’ stronger identity centrality and body appreciation.

Finally, as is the case with most questionnaire studies, the study is based on self-reports. Even though participants who might have given biased responses in the form of inattentive responses were excluded from the study [59, 60], biased responses might have resulted from participants’ different levels of ability to self-reflect on their behaviors and experiences.

## Conclusion

The current study replicated findings that report lower levels of body appreciation in sexual minority individuals in comparison to heterosexual persons [6, 116, 117] and lower levels of body appreciation in non-binary persons as compared to cisgender men [29, 118]. The study adds to the literature by showing that the social cure model [11] might explain associations between lower levels of in-group pressure to adhere to cis-heteronormative body standards and higher levels of body appreciation in non-binary persons, or gay/lesbian persons and asexual persons. However, bisexual/pansexual cisgender men experienced higher levels of in-group pressure than did heterosexual men which is in line with findings that bisexual/pansexual persons can experience discrimination from heterosexual individuals and/or sexual minority individuals [92, 95].

The current study adds to the scarce literature about non-binary persons’ experiences of hostility directed towards them because of their looks or body that might result because of not conforming to cis-heteronormative body standards [5, 26, 89]. Future research about identity centrality, in-group pressure, experiences of hostile behaviors, and body appreciation needs to include transgender persons and considerations of medical or non-medical gender-affirming intervention or body modifications [29].

## Electronic supplementary material

Below is the link to the electronic supplementary material.

Supplementary Material 1

## Data Availability

The datasets used and/or analyzed in the present study are available from the corresponding author on reasonable request.

## Footnotes

1 A partial correlation controlling for gender, sexual orientation, nationality, education, employment, age, BMI shows that the recruitment method did not correlate with any variable of interest in the study (Supplemental Material S1).

## Declarations

### Previous presentations

No previous presentations.
