# Supplementary material for: Social cure model: testing the link between identity centrality and body appreciation in diverse sexual orientation and gender identity groups
Source: Int J Equity Health. 2024 Sep 18;23:185. doi: 10.1186/s12939-024-02268-3 (PMC11409622; doi:10.1186/s12939-024-02268-3)
Supplement: Supplementary file 1 — Supplementary Material 1 [file 12939_2024_2268_MOESM1_ESM.docx]

# Supplemental Material

### S1

Partial correlation testing whether the recruitment method correlated with the variables of interest while controlling for sociodemographic variables (gender, sexual orientation, nationality, education, employment, age, BMI)

| Variable | Hostile behavior | Identity centrality | Pressure in-group | Body appreciation |
| --- | --- | --- | --- | --- |
| 1. Source (e-Mail, Facebook/Instagram, Prolific, other) | -.02 | -.03 | .01 | -.03 |

### S2

List of and responses to sexual orientation identity labels, gender identity labels, intersection of sexual orientation identity labels and gender identity labels, or less specific social group labels

| Response option | *N* (%) |
| --- | --- |
| Woman | 272 (17.1) |
| Man | 180 (11.3) |
| Heterosexual man | 175 (11.0) |
| Queer | 135 (8.5) |
| Heterosexual woman | 120 (7.6) |
| LGBTQ* person | 119 (7.5) |
| General population | 102 (6.4) |
| Bisexual woman | 99 (6.2) |
| Cis woman | 89 (5.6) |
| Gay man | 60 (3.8) |
| Cis man | 58 (3.7) |
| Lesbian woman | 37 (2.3) |
| Bisexual man | 21 (1.3) |
| Gender-neutral/non-binary person | 20 (1.3) |
| Pansexual woman | 19 (1.2) |
| Asexual woman | 17 (1.1) |
| Asexual person | 12 (0.8) |
| Sexual and gender minority | 9 (0.6) |
| Heterosexual person | 7 (0.4) |
| Bisexual person | 6 (0.4) |
| Non-heterosexual trans* person | 6 (0.4) |
| Pansexual man | 5 (0.3) |
| Pansexual person | 5 (0.3) |
| Asexual man | 4 (0.3) |
| Transgender person | 4 (0.3) |
| Gay/lesbian person | 3 (0.2) |
| Trans* men | 3 (0.2) |

### S3

Descriptive Statistics by Gender x Sexual Orientation

|  | Cisgender man x heterosexual | Cisgender man x gay | Cisgender man x bi/pansexual | Cisgender man x asexual | Cisgender woman x heterosexual | Cisgender woman x lesbian | Cisgender woman x bi/pansexual | Cisgender woman x asexual | Non-binary x heterosexual | Non-binary x gay/lesbian | Non-binary x bi/pansexual | Non-binary x asexual |
| --- | --- | --- | --- | --- | --- | --- | --- | --- | --- | --- | --- | --- |
| Hostile behavior | 1.5 (0.6) | 1.7 (0.7) | 1.7 (0.8) | 1.6 (0.6) | 1.5 (0.6) | 1.5 (0.7) | 1.6 (0.7) | 1.6 (0.7) | 1.8 (0.9) | 2.0 (0.8) | 1.7 (0.6) | 1.7 (0.7) |
| Pressure in-group | 2.0 (0.9) | 2.2 (1.0) | 2.7 (1.0) | 1.9 (0.9) | 2.4 (1.0) | 1.9 (0.9) | 1.9 (0.9) | 1.6 (0.7) | 2.2 (1.0) | 1.8 (0.8) | 2.3 (1.0) | 1.7 (0.8) |
| Identity centrality | 2.9 (1.1) | 2.8 (1.1) | 3.7 (0.9) | 2.8 (1.1) | 3.3 (1.0) | 3.5 (1.0) | 3.9 (0.9) | 3.4 (1.0) | 3.0 (0.9) | 3.9 (0.9) | 4.3 (0.6) | 4.1 (0.7) |
| Body appreciation | 3.5 (0.8) | 3.2 (0.8) | 3.3 (0.8) | 3.2 (0.9) | 3.5 (0.8) | 3.4 (0.8) | 3.3 (0.9) | 3.3 (0.8) | 3.5 (1.1) | 3.1 (0.8) | 3.4 (0.7) | 2.9 (0.7) |
